# Muscle ultrasound for early assessment of critical illness neuromyopathy in severe sepsis

**DOI:** 10.1186/cc13050

**Published:** 2013-10-07

**Authors:** Alexander Grimm, Ulrike Teschner, Christine Porzelius, Katrin Ludewig, Jörg Zielske, Otto W Witte, Frank M Brunkhorst, Hubertus Axer

**Affiliations:** 1 Hans Berger Department of Neurology, Jena University Hospital, Erlanger Allee 101, D-07747 Jena, Germany; 2 Center for Sepsis Control and Care (CSCC), Jena University Hospital, Erlanger Allee 101, D-07747 Jena, Germany; 3 Institute for Medical Informatics, Statistics and Epidemiology (IMISE), University Leipzig, Härtelstr. 16-18, D-04107 Leipzig, Germany; 4 Department of Otolaryngology, Jena University Hospital, Lessingstr. 2, D-07743 Jena, Germany; 5 Paul-Martini-Clinical Sepsis Research Unit, Department of Anaesthesiology and Intensive Care Medicine, Jena University Hospital, Erlanger Allee 101, D-07747 Jena, Germany

## Abstract

**Introduction:**

Muscle ultrasound is emerging as a promising tool in the diagnosis of neuromuscular diseases. The current observational study evaluates the usefulness of muscle ultrasound in patients with severe sepsis for assessment of critical illness polyneuropathy and myopathy (CINM) in the intensive care unit.

**Methods:**

28 patients with either septic shock or severe sepsis underwent clinical neurological examinations, muscle ultrasound, and nerve conduction studies on days 4 and 14 after onset of sepsis. 26 healthy controls of comparable age underwent clinical neurological evaluation and muscle ultrasound only.

**Results:**

26 of the 28 patients exhibited classic electrophysiological characteristics of CINM, and all showed typical clinical signs. Ultrasonic echogenicity of muscles was graded semiquantitatively and fasciculations were evaluated in muscles of proximal and distal arms and legs. 75% of patients showed a mean echotexture greater than 1.5, which was the maximal value found in the control group. A significant difference in mean muscle echotexture between patients and controls was found at day 4 and day 14 (both p < 0.001). In addition, from day 4 to day 14, the mean grades of muscle echotexture increased in the patient group, although the values did not reach significance levels (p = 0.085). Controls revealed the lowest number of fasciculations. In the patients group, fasciculations were detected in more muscular regions (lower and upper arm and leg) in comparison to controls (p = 0.08 at day 4 and p = 0.002 at day 14).

**Conclusions:**

Muscle ultrasound represents an easily applicable, non-invasive diagnostic tool which adds to neurophysiological testing information regarding morphological changes of muscles early in the course of sepsis. Muscle ultrasound could be useful for screening purposes prior to subjecting patients to more invasive techniques such as electromyography and/or muscle biopsy.

**Trial registration:**

German Clinical Trials Register, DRKS-ID: DRKS00000642.

## Introduction

Critical illness polyneuropathy (CIP) and critical illness myopathy (CIM) as well as the combination of both are common sequelae in patients with severe sepsis in ICUs [1-3]. The common clinical feature of unclassified CIM and CIP comprises ICU-acquired weakness (ICUAW). About 70% of patients with severe sepsis develop alterations of the peripheral nervous system [4,5]. Whereas symmetric distally predominant muscle weakness with atrophy, loss of deep tendon reflexes, and often a distal reduction of sensitivity to pain, temperature, and vibration are typical clinical features of CIP [6], early failure of weaning from the ventilator and proximal muscle weakness are more suggestive of CIM. Development of CIP contributes to longer ventilator time and a prolonged in-hospital stay [5,7]. Moreover, CIP is associated with increased in-hospital mortality [8].

The current gold standard for diagnosis of CIP and CIM in the ICU setting consists of a careful neurological examination together with nerve conduction studies and electromyography (EMG) [9]. Typical electroneurographic signs of CIP are reductions of the amplitude of compound muscle action potentials as well as sensory nerve action potentials [10,11]. In particular, the decrease of compound motor action potential develops within 2 to 5 days [12]. However, there is no direct relationship between ICUAW and electrophysiological (muscle or nerve) abnormalities with a high prevalence of electrophysiological abnormalities early on in critical illness. In addition, assessment of Medical Research Council scores of muscle strength and classical EMG examinations beyond the detection of spontaneous activity often cannot reliably be performed since, in the acute phase of severe sepsis, patients are as a rule unconscious and hence uncooperative.

Yet, early detection of critical illness neuromyopathy is beneficial for improving standards of care [1,13,14]. Accordingly, there is a pressing need for easily applicable and non-invasive instruments for evaluation of muscle state in critically ill patients.

Muscle ultrasound has shown growing promise in the diagnosis of neuromuscular diseases [15-18]. Axonal nerve damage in CIP and direct muscle impairment in CIM cause changes in muscle structure as well as induce spontaneous muscle activity [1,2]. Both structural changes and spontaneous activity are detectable using muscle ultrasound.

We performed a first feasibility study in patients with electrophysiological proven critical illness neuromyopathy and clinical signs of ICUAW to evaluate whether muscle ultrasound allows visualization of changes in the muscle echotexture during the early course of sepsis.

## Materials and methods

### Patients

Between October 2011 and August 2012 we prospectively performed standardized muscle ultrasound examinations in patients with either severe sepsis or septic shock. All patients were enrolled in the NeuroSOS-NERVE Study (German Clinical Trials Register [DRKS-ID:DRKS00000642]). The study is still ongoing and focuses on the impairment of small sensory nerve fibers in skin biopsies. The study has been approved by the local ethics committee (Ethics Commission of the Friedrich-Schiller-University Jena, No. 2771-02/10). Written informed consent was obtained from all patients or their legal representative.

Severe sepsis and septic shock were defined according to published criteria [19,20]. Patient eligibility was screened daily by trained ICU research nurses. Exclusion criteria comprised a history of neuromuscular disorders (for example, polyneuropathy, myopathy, motor neuron disease, and others), known alcohol abuse, high-dose steroid therapy before sepsis. Furthermore, we excluded patients with protracted critical illness – that is, an ICU stay >8 days prior to enrollment – to ensure a more homogeneous population with acute critical illness.

All patients were examined between days 2 and 5 (visit 1) and on day 14 (visit 2) after the onset of severe sepsis or septic shock, respectively. Each patient received a clinical neurological examination including evaluation of Medical Research Council muscle strength and Rankin score, and underwent muscle ultrasound as well as nerve conduction studies. The same clinical neurological examination and muscle ultrasound protocol were undertaken in a group of healthy controls of comparable age.

### Ultrasound protocol

Ultrasonography was performed using a 9 to 13 MHz probe real-time linear array scanner (Siemens Acuson, Erlangen, Germany). The initial settings (as contrast) were kept constant during all examinations excluding the depth, which was altered individually to visualize the complete muscle. Performers of ultrasound examination were blinded to the clinical and electrophysiological parameters. Ultrasonography was performed bilaterally in different muscles of the upper and lower limbs. Patients were examined in the supine position with extended arms and legs and relaxed muscles [21,22]. The muscles were scanned in both axial and longitudinal planes, and each muscle was evaluated at standardized anatomical points (in detail: biceps brachii and quadriceps femoris muscles at the midline between origin and insertion, the extensor muscles of the forearms at the first third of the distance between the elbow and processus styloideus radii, and the tibialis anterior muscle at the first third between the knee and malleolus lateralis).

**Figure 1:**
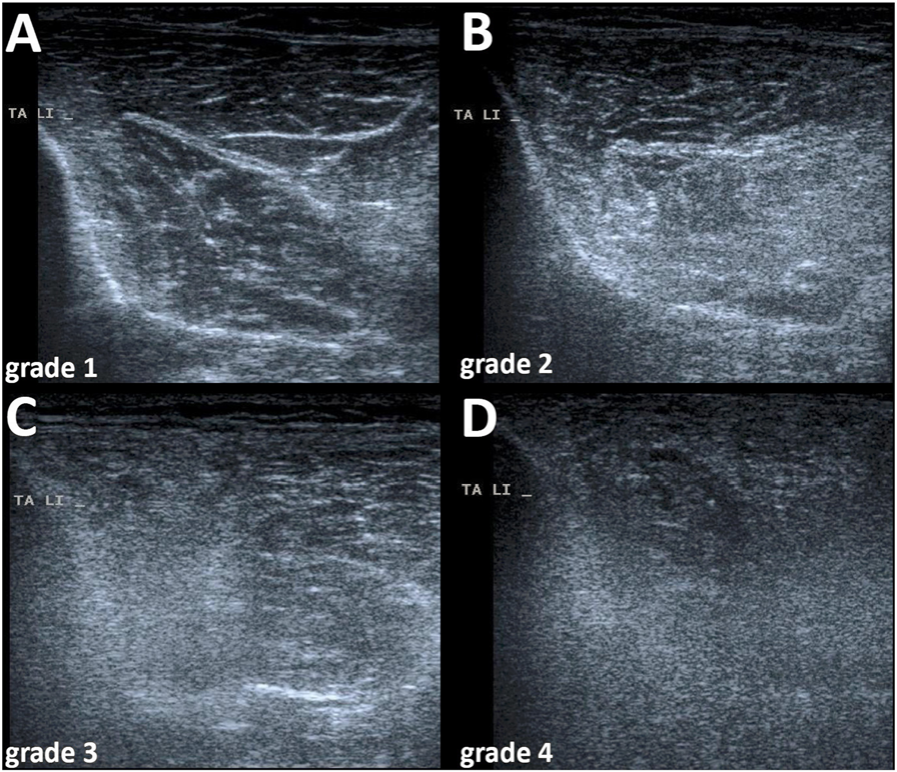
**Ultrasonic cross-sections through the tibialis anterior muscles showing different grades in echogenicity as defined by the Heckmatt score**[23]**. (A)** Normal echo intensity with starry-night aspect with distinct bone echo in a healthy control. **(B)** Increased echo intensity with normal bone echo in a septic patient at day 4. **(C)** Increased echo intensity with reduced bone signal in a septic patient at day 14. **(D)** Increased echo intensity and loss of bone signal in a septic patient at day 14.

Assessment of ultrasonic muscle echogenicity was one of the key parameters of the study. Compression of the tissue and oblique scanning were avoided because of the risk of an artificial change in muscle echotexture. Ultrasonic echogenicity was graded according to Heckmatt and colleagues [23]. This score differentiates ultrasonic echogenicity semiquantitatively into four grades (Figure 1). A higher grade of echotexture with reduced or lost bone signal correlates to the severity of muscle impairment.

Ultrasonic detection of muscle fasciculations was the second key parameter. Each muscle was scanned over a time period of 10 seconds at three different measurement points without moving the probe, as described previously [24,25]. Fasciculations are visible as short twitches of bundles of muscle fibers. They were classified as spontaneous activity if they appeared at least twice. Fasciculations can easily be distinguished from other movements such as arterial pulsations (unifocal, rhythmic) or voluntary movements (involving the entire muscle) [26]. Analysis of ultrasound data was performed both online and offline. Complete ultrasonographic examination of each patient necessitated about 25 minutes.

### Nerve conduction studies

Additionally, standardized nerve conduction studies were carried out using a portable electro-neurophysiologic device (Synergy 15.0; VIASYS Healthcare UK Ltd, CareFusion Germany, Hoechberg, Germany). The right median nerve, the right tibial nerve, the left fibular nerve, and both sural nerves were measured. Motor and sensory nerve responses were assessed for the right median nerve, motor responses only for the tibial and fibular nerve, and sensory nerve responses for both sural nerves. Electroneurographic criteria for the diagnosis of CIP were a reduction in the amplitude of compound muscle action potentials and sensory nerve action potentials [10,27].

### Statistical analysis

A grading of overall echotexture was calculated as the mean of the grades of echotexture analyzed in the four different regions (proximal and distal arm, proximal and distal leg). To compare the mean echotexture of controls and patients (day 4 and 14), Wilcoxon rank-sum tests were used. For the comparison of the mean echotexture of patients at day 4 and 14, Wilcoxon signed-rank tests were applied because samples are paired. Patients without measurements at day 14 due to death were thus excluded. The Cochran–Armitage test for trend was applied for comparison of the number of regions with detected fasciculations between healthy controls and patients (measurements at days 4 and 14). Since this study had an exploratory character, no adjustment for multiple testing was performed.

## Results

### Baseline characteristics of patients

A total of 28 patients with sepsis (21 with septic shock, seven with severe sepsis) were enrolled (median age 69.5 years, interquartile range 61.5 to 75.25 years; 25 male, three female). Six patients died before the second visit at day 14. Furthermore, 26 healthy controls (median age 64 years, interquartile range 61 to 76 years; 12 male, 14 female) were also examined.

The baseline characteristics of the patients and microbiological findings are presented in Tables 1 and 2. None of the patients was exposed to risk factors for CIM, such as high-dose steroids or neuromuscular blocking agents (exclusion criteria of the study). All patients had lost or at least attenuated muscle reflexes and also showed significant muscle weakness (Table 3). Twenty of the 28 patients (71%) were mechanically ventilated at the first visit and 10 out of 22 patients (45.5%) at the second visit. Most of the patients received vasopressors, sedative drugs, and opioids (Table 3).

**Table 1:** **Demographics and baseline characteristics of patients (*n *** **= 28)**

Age (years)	69.5 (61.5 to 75.25)
Male gender	25 (89.29%)
Body mass index^a^	27.7 ± 7.1
APACHE II score^b^	22.5 ± 6.5
Septic shock	21 (75%)
Duration of ventilation (days)	20.5 ± 13.7
Days of stay on ICU	35.1 ± 71.3
Days of stay on ICU before sepsis	3.0 ± 4.7
Renal replacement therapy	12 (42.86%)
Days with renal replacement therapy	4.4 ± 7.8
Days with vasopressors	11.14 ± 9.55
Days with sedation	8.9 ± 8.7
Cumulative doses	
Midazolam (mg)	6,930.3 ± 1,153.8
Propofol (mg)	10,784.8 ± 13,749.6
Ketamine (mg)	11,52.1 ± 3,347.1
Clonidine (μg)	1,506.4 ± 4,118.7
Days with opioids	8.9 ± 8.8
Cumulative doses	
Sufentanyl (mg)	14.9 ± 25.0
Morphine (mg)	233.0 ± 147.9
Preexisting conditions^c^	
History of diabetes	9 (32.1%)
Heart failure	7 (25.0%)
Cerebrovascular disease	10 (35.7%)
Renal dysfunction	4 (14.3%)
Chromic obstructive pulmonary disease	4 (14.3%)
Liver cirrhosis	1 (3.6%)
History of cancer	3 (10.7%)
Immunosuppression	2 (7.1%)
Recent surgical history	
Elective surgery	1 (3.6%)
Emergency surgery	6 (21.4%)
No history of surgery	21 (75.0%)
Site of infection	
Pneumonia	13 (46.4%)
Abdomen	6 (21.4%)
Tracheobronchitis	3 (10.7%)
Urosepsis	3 (10.7%)
Meningitis	1 (3.6%)
Wound infection	1 (3.6%)
Cerebral empyema	1 (3.6%)
Hospital mortality	10 (35.7%)

Data presented as median, (interquartile range), *n* (%) or mean ± standard deviation. APACHE, Acute Physiology and Chronic Health Evaluation. ^a^Calculated as weight in kilograms divided by height in meters squared.

^b^Missing subscores were counted as 0. The scale score range is from 0 to 71, with higher scores indicating a greater severity of illness.

^c^Multiple entries per patient possible.

**Table 2:** **Microbiological findings**^**a**^

	**Material**						
	**Blood culture**	**Tracheal secretion**	**Bronchoalveolar lavage**	**Pleural puncture**	**Peritoneal puncture**	**Cerebrospinal fluid**	**Total**
*Staphylococcus aureus*	0	1	1	0	1	0	3
Coagulase-negative staphylococcus species	1	0	0	0	0	0	1
Enterococci	0	0	8	0	1	0	9
Streptococci group A to C, G	0	0	0	0	1	0	1
*Streptococcus pneumoniae*	0	0	0	0	0	1	1
Other streptococci	1	0	0	2	0	0	3
Other aerobic gram-positive bacteria	1	0	0	0	0	0	1
*Escherichia coli*	2	0	1	2	2	0	7
Enterobacter	0	0	1	0	0	0	1
*Klebsiella*	3	0	1	1	0	0	5
*Proteus* spp.	0	0	1	0	0	0	1
Other gram-negative bacteria	0	0	1	0	0	0	1
total	8	1	14	5	5	1	34

^a^Multiple entries per patient possible.

**Table 3:** Neurological characteristics of patients at the two visits

**Visits**	**Day 4**		**Day 14**	
Number of patients	28		22	
Mechanical ventilation	20 (71.4%)		10 (45.5%)	
Mean cumulative fluid balance	In: 21,935 ml		In: 66,028 ml	
	Out: –16,533 ml		Out: –64,969 ml	
	Net: 5,402 ml		Net: 1,059 ml	
Rankin score	Rankin score	Number	Rankin score	Number
	2	0 (0%)	2	1 (4.6%)
	3	3 (10.7%)	3	2 (9.1%)
	4	2 (7.1%)	4	4 (18.2%)
	5	23 (82.1%)	5	12 (68.2%)
Muscle strength proximal arm	MRC score	Number	MRC score	Number
	0 to 1	18 (64.3%)	0 to 1	9 (40.9%)
	2	4 (14.3%)	2	2 (9.1%)
	3	3 (10.7%)	3	6 (27.3%)
	4	3 (10.7%)	4	4 (18.2%)
	5	0 (0%)	5	1 (4.5%)
Muscle strength distal arm	MRC score	Number	MRC score	Number
	0 to 1	20 (71.4%)	0 to 1	9 (40.9%)
	2	1 (3.6%)	2	2 (9.1%)
	3	1 (3.6%)	3	5 (22.7%)
	4	4 (14.3%)	4	5 (22.7%)
	5	2 (7.1%)	5	1 (4.5%)
Muscle strength proximal leg	MRC score	Number	MRC score	Number
	0 to 1	19 (67.9%)	0 to 1	8 (36.4%)
	2	4 (14.3%)	2	5 (22.7%)
	3	2 (7.1%)	3	4 (18.2%)
	4	3 (10.7%)	4	4 (18.2%)
	5	0 (0%)	5	0 (0%)
Muscle strength distal leg	MRC score	Number	MRC score	Number
	0 to 1	19 (67.9%)	0 to 1	10 (45.5%)
	2	2 (7.1%)	2	2 (9.1%)
	3	2 (7.1%)	3	2 (9.1%)
	4	5 (17.9%)	4	6 (27.3%)
	5	0 (0%)	5	2 (9.1%)
Biceps reflex		Number		Number
	Absent	15 (53.6%)	Absent	13 (59.1%)
	After reinforcement	3 (10.7%)	After reinforcement	3 (13.6%)
	Attenuated	10 (35.7%)	Attenuated	5 (22.7%)
	Normal	0 (0%)	Normal	1 (4.5%)
Patellar reflex		Number		Number
	Absent	18 (64.3%)	Absent	9 (40.9%)
	After reinforcement	4 (14.3%)	After reinforcement	2 (9.1%)
	Attenuated	6 (21.4%)	Attenuated	10 (45.5%)
	Normal	0 (0%)	Normal	1 (4.5%)
Ankle jerk reflex		Number		Number
	Absent	26 (92.9%)	Absent	18 (81.8%)
	After reinforcement	1 (3.6%)	After reinforcement	1 (4.5%)
	Attenuated	1 (3.6%)	Attenuated	2 (9.1%)
	Normal	0 (0%)	Normal	1 (4.5%)
Patients with drugs	Sufentanyl	15 (53.6%)	Sufentanyl	8 (36.4%)
	Midazolam	6 (21.4%)	Midazolam	4 (0.2%)
	Propofol	6 (21.4%)	Propofol	0 (0%)
	Clonidine	6 (21.4%)	Clonidine	1 (0.05%)
	Vasopressors	20 (71.4%)	Vasopressors	8 (36.4%)

MRC, Medical Research Council.

### Nerve conduction studies

Nerve conduction studies were performed in 26 patients at day 4 and in 18 patients at day 14. All patients exhibited the typical electrophysiological characteristics of CIP – that is, reductions of amplitudes of compound motor action potential and sensory nerve action potential (see Additional file 1) – in contrast to relatively small reductions in nerve conduction velocity in at least three of the nerves measured. Local reference values were used as lower limits for electroneurography amplitudes. All patients measured thus suffered from CIP according to electroneurographic criteria.

### Ultrasonic muscle echogenicity

Ultrasonic echogenicity was graded semiquantitatively according to Heckmatt and colleagues [23]. Figure 1 shows some examples of different grades of echotexture in the tibialis anterior muscle. Interimage measurements demonstrated an intraclass correlation coefficient of 0.915 between raters, while the intrarater intraclass correlation coefficient was 0.972.

**Figure 2:**
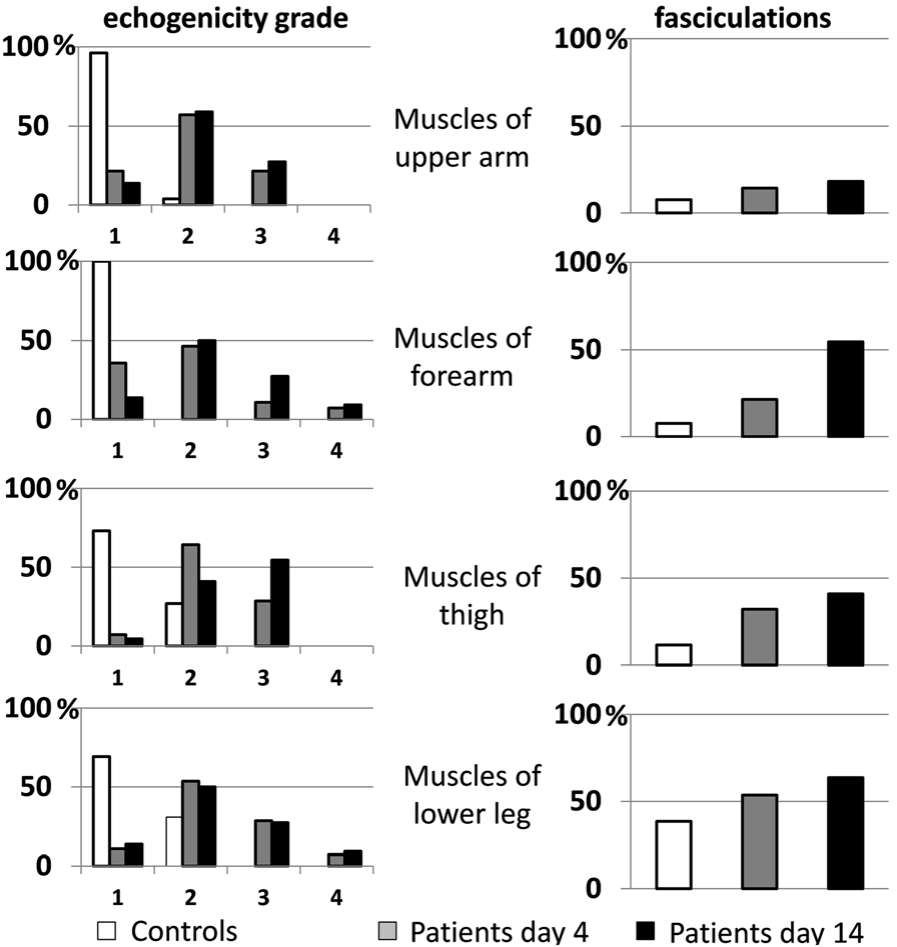
**Frequency of ultrasonographic echogenicity grades and detection of fasciculations.** Frequency (%) of different ultrasonographic echogenicity grades as defined by the Heckmatt score [23] and detection of fasciculations in different anatomical regions for patients at day 4 and day 14 and for controls.

**Figure 3:**
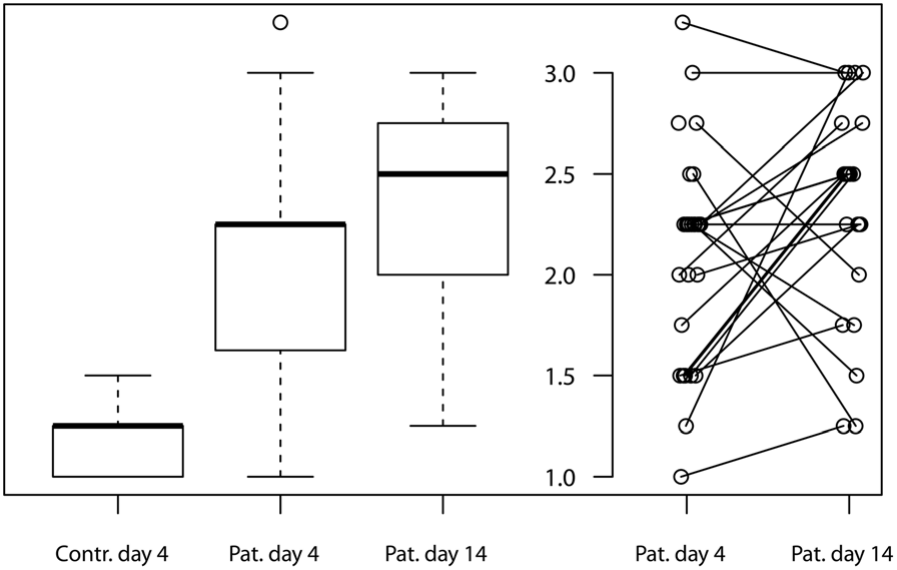
**Boxplots of mean echotexture over four anatomical regions.** For patients, additional parallel coordinate plots are shown to illustrate differences over time. Each patient is represented by a grey circle. Contr., control; Pat., patient.

Figure 2 illustrates the distribution of muscle echotexture and fasciculations in controls and patients at day 4 and day 14 separately in the four anatomical regions analyzed. All echotexture scores of the four muscle regions examined were averaged (Figure 3) to generate a mean echotexture score. Ninety-two percent of the controls revealed a normal mean echotexture (grade 1 or 1.25). Seventy-five percent of the patients showed a mean echotexture greater than 1.5, which was the maximal value found in the control group. A significant difference in mean muscle echotexture between patients and controls was found at day 4 and day 14 (both *P* <0.001). In addition, the mean grades of muscle echotexture in the patient group increased between day 4 and day 14, but did not reach statistical significance (*P* = 0.085).

To avoid averaging the semiquantitative scores of echotexture over all muscles analyzed, single trend tests per visit and anatomical muscle region were performed separately and all Cochran–Armitage tests for trend were significant (*P* <0.001, not shown in detail).

### Ultrasonic detection of fasciculations

Fasciculations were sonographically detected in different anatomical regions in the patient group, but also in 38% in the distal leg muscles of the healthy controls (Figure 2). In all anatomical regions analyzed, healthy controls revealed the lowest number of detected fasciculations, whilst patients at day 14 had more fasciculations than patients at day 4 as fasciculations increased over time of sepsis.

A fasciculation score that ranged from 0 to 4 and simply counted the anatomical regions with ultrasonically detected fasciculations was conceived (that is, 0 to 4 regions with fasciculations out of four muscle regions). The Cochran–Armitage test for trend for comparing healthy controls versus patients revealed *P* = 0.080 at day 4 (Table 4) and *P* = 0.002 at day 14 (Table 5).

**Table 4:** Number of muscle regions with fasciculations in proximal and distal upper and lower extremities in patients at day 4 versus controls

	**Number of muscle regions with fasciculations**					**Total**
	**0/4**	**1/4**	**2/4**	**3/4**	**4/4**	
Patients	10	9	5	1	3	28
Controls	15	8	1	1	1	26
Total	25	17	6	2	4	54

Cochran–Armitage test for trend: χ^2^ = 3.056, degrees of freedom = 1, *P* = 0.080.

**Table 5:** Number of muscle regions with fasciculations in proximal and distal upper and lower extremities in patients at day 14 versus controls

	**Number of muscle regions with fasciculations**					**Total**
	**0/4**	**1/4**	**2/4**	**3/4**	**4/4**	
Patients	4	6	5	5	2	22
Controls	15	8	1	1	1	26
Total	19	14	6	6	3	48

Cochran–Armitage test for trend: χ^2^ = 9.590, degrees of freedom = 1, *P* = 0.002.

### Regional and anatomical relationships

In addition, a weak correlation was found between the mean echogenicity score and the fasciculation score at both day 4 (Pearson correlation coefficient 0.399, *P* = 0.001) and day 14 (Pearson correlation coefficient 0.615, *P* <0.001).

Comparison of echogenicity between proximal and distal muscles in the patient group did not reach significance at both visits, while detected fasciculations differed significantly between proximal and distal muscles of the arm and the leg at day 14 (McNemar’s test for paired samples, *P* = 0.008 each). Fasciculations were detected more frequently in the distal than in the proximal muscles in the patients at day 14.

**Figure 4:**
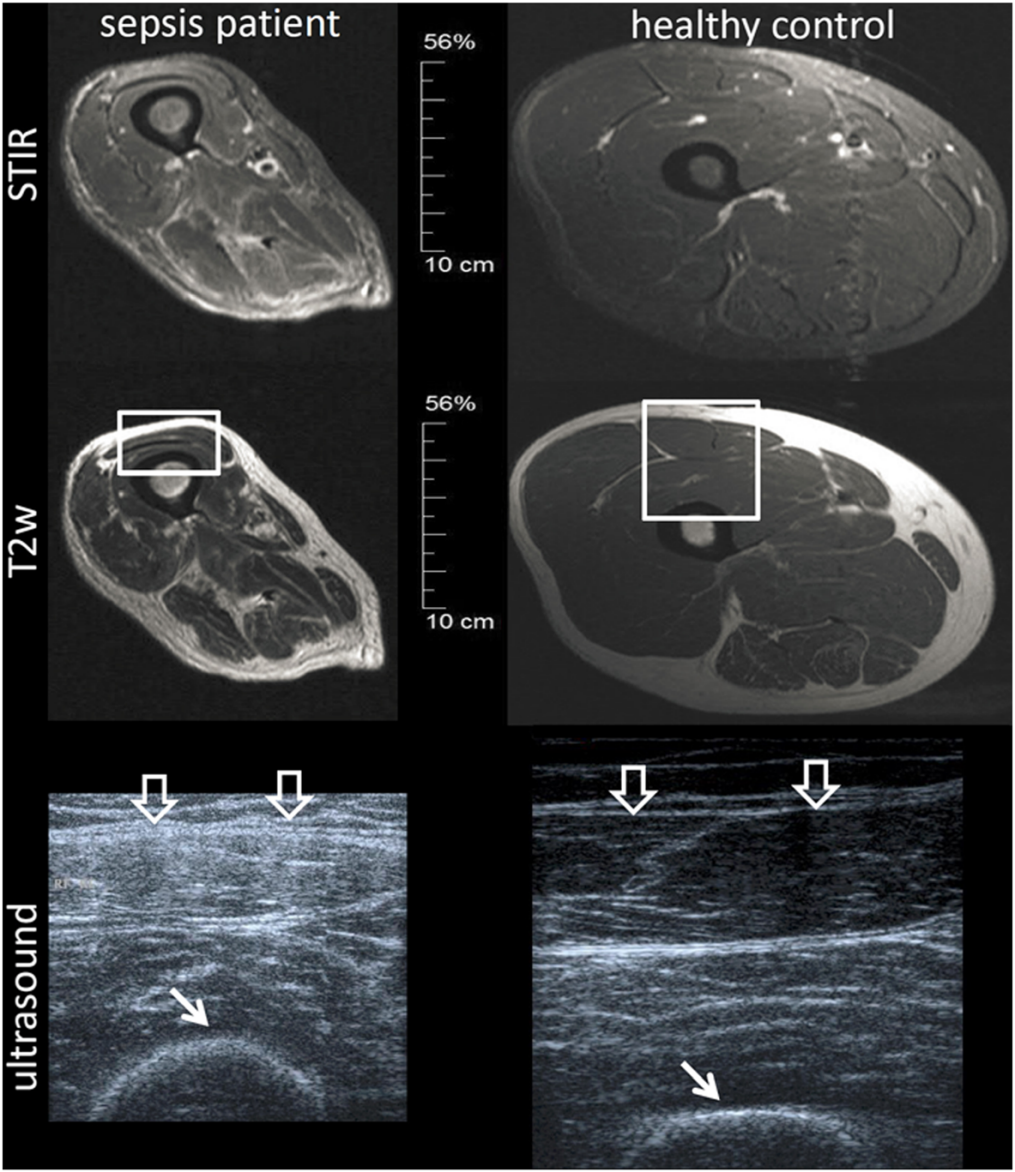
**Muscle magnetic resonance imaging (1.5 Tesla) of a sepsis patient and a healthy control.** Left: sepsis patient at day 14. Right: healthy control. Short TI inversion recovery (STIR) is a magnetic resonance imaging (MRI) sequence to suppress the signal from fat, so that especially edema can be seen in the muscle. The images demonstrate that at day 14 after onset of severe sepsis the structural changes in the muscle (atrophic and fibrous changes) dominate over edematous changes. Ultrasonic sections of the same subjects through the rectus femoris muscle (large arrows) are shown at the bottom. The corresponding areas in MRI are marked as rectangles. The difference in echogenicity of the muscle can clearly be seen. Moreover, the bone signal in the patient begins to blur (small arrows).

Figure 4 depicts a magnetic resonance imaging scan (1.5 Tesla) of the thigh in a sepsis patient at day 14 in comparison with a healthy control, to illustrate structural changes of the muscles seen in ultrasound compared with those seen in magnetic resonance imaging.

## Discussion

Results of this first pilot study suggest a promising role for bedside ultrasound as an additive non-invasive procedure for detecting changes in muscle architecture in patients with either septic shock or severe sepsis. Several studies have demonstrated that muscle ultrasound is able to reliably detect pathological changes [15-18] such as muscle atrophy, fatty infiltration, and intramuscular fibrosis [28,29]. The combination of static and dynamic images in B-mode and M-mode [30,31] enables the detection of spontaneous muscle activity, so that muscle fasciculations can be visualized with higher sensitivity by ultrasound compared with EMG measurements [14,24,31].

### Limitations of the study

Our study has several limitations. First, patient numbers were small and patient age was relatively high. Second, one-third of the patients had coexistent diabetes mellitus, which may enhance susceptibility for neuropathic disorders such as diabetic polyneuropathy. However, preexisting known neuropathy or myopathy were exclusion criteria for the study. Third, patients and controls were not sex-matched as we did not focus on sex-dependent differences herein. Finally, the timeframe for initial assessment (that is, 2 to 5 days after onset of sepsis) was relatively broad and the ICU stay before onset of severe sepsis varied between patients, which may have contributed to alterations in the muscle state between study visits. Ultrasound characteristics were restricted to semiquantitative grading of echogenicity and the detection of fasciculations. Additional parameters such as atrophy of the muscles that can be evaluated by diameter, area, and volumes of the muscles measured in ultrasonic cross-sections were not considered.

Echotexture grading using a semiquantitative scale may be subject to technical misinterpretation in contrast to objective, user-independent algorithms for image analysis [21,26]. However, the Heckmatt score used in our study is a rapid applicable bedside technique that can be easily used in the intensive care setting. Moreover, intrarater and interrater reliability measurements in this study were good and did not differ from the interrater and intrarater intraclass correlation coefficients of muscle ultrasound measurements described in the literature [32,33].

### Muscle echogenicity

We found significant alterations in muscle echotexture in the early stage of sepsis compared with healthy controls. Similar changes have also been described in a recent study of 16 patients with acute respiratory distress syndrome [34]. The marked changes in muscle echotexture during sepsis may refer to muscle edema due to capillary leak in the acute stage and to fibrosis and fatty degeneration in the subacute stage of sepsis.

Excessive fluid resuscitation with positive fluid balance in septic shock patients may partly account for unspecific muscle edema in the acute stage of sepsis. Changes in superficial tissues due to edema may affect the interpretation of muscle images and may also cause artificial amplitude reductions in nerve conduction measurements. However, in the later stages of severe sepsis (that is, on day 14 in our study), fluid balances are more balanced [35].

In our study, mean cumulative fluid balances at day 4 and day 14 were positive (5.4 l and 1 l respectively; see Table 3). Since different tissues may lose fluid at different rates, it is difficult to clarify from our data whether muscle echogenicity may be caused by edema or genuine muscle impairment. However, the significance of tissue edema in the assessment of muscle echogenicity may be overestimated, since tissue edema cannot alter the bone signal in contrast to fibrous tissue, which also contributes to the grading in the Heckmatt score [36]. It has been shown earlier that ultrasonic echogenicity scores mainly correlate to fibrous tissue content in muscle biopsies [28], and the detection of fasciculations *per se* cannot be altered by edema.

Since the muscle echotexture score increased in our study from day 4 to day 14 in the presence of decreasing fluid balances at day 14, a specific structural damage in muscle architecture can be assumed. This is supported by findings from muscle magnetic resonance imaging in a patient on day 14 showing significant changes in muscle architecture without tissue edema (Figure 4).

Structural muscle changes can be due to muscle wasting [37], which may be caused by immobility, but also by direct muscle impairment in CIM and indirect muscle impairment in CIP, due to denervation [38]. Muscle wasting may enhance echogenicity due to a relative increase of fibrous tissue caused by a decrease of muscle fiber volume. The proportion of myopathy, neuropathy, and immobility-induced atrophy could not be definitely proven in our study. Nerve conduction studies, however, revealed that the majority of our patients suffered from CIP, since motor as well as sensible nerve fibers were impaired according to electrophysiological evidence. The additional existence of CIM could not been ruled out, as CIP and CIM often co-occur [14,39], and muscle biopsies or direct muscle stimulation [40,41] were not performed to distinguish CIP from CIM.

### Fasciculations

Our study suggests that ultrasound is also capable of detecting muscle fasciculations in the early course of sepsis. Muscle fasciculations represent spontaneous activity in the muscle, which is a sign of increased excitability of the impaired motor nerves. Although fasciculations have not been examined using EMG in our study, it is well established that detection of fasciculations is more sensitive using ultrasound than by EMG, because a larger muscle area can be analyzed [24,42].

Previously it has been shown that the median time to develop spontaneous activity in EMG is 21 days after ICU admission [43]. In contrast, we observed muscle fasciculations after only 4 days. Such early changes might be more likely to arise in CIM [44]. Since the mean length of ICU stay before onset of sepsis was 3 days (see Table 1), many patients were already critically ill before enrollment. This may also account for the 'relatively’ early detection of fasciculations in the muscles because denervation may have already been present before severe sepsis as sepsis is not the only risk factor for CIMN.

Although more fasciculations were detectable in septic patients at day 4 compared with controls, the difference did not reach statistical significance. Furthermore, fasciculations were also apparent in a considerable number of controls. This may be due to pre-existing subclinical nerve lesions in older subjects in the control group; for example, caused by prevailing radiculopathy (L5 and S1) due to lumbar disc degeneration most notably in the distal leg.

Nevertheless, in the patient group fasciculations increased significantly over different muscle regions over time. The increased involvement of arm muscles over time may be a sign of sepsis-induced muscle alteration.

There was a weak correlation between mean echogenicity score and fasciculation score, which points to an interrelationship between functional signs of axonal denervation and changes of muscle structure.

### Link to function

Electrophysiological changes in critically ill patients may evolve early and also be rapidly reversible [1], so that these changes do not directly reflect functional outcome of the patients. This is also due to excitability changes in muscle and axonal membranes, which cause early functional impairments before structural damage occurs [38]. At this early time point ICUAW can be reversible, as is seen by the fact that many patients recover well.

In our study neither nerve conduction studies nor muscle ultrasound correlated significantly with function (that is, Medical Research Council scores, Rankin score, weaning times, and so forth; not shown in detail). However, our patient group consisted of severely ill patients many of whom were mechanically ventilated and sedated, and hence clinical muscle strength could not be reliably measured and follow-up examinations of functional impairments were not performed. More information is therefore needed to correlate functional outcome over a longer follow-up period in association with ultrasonic measurements.

## Conclusions

In summary, we present a first feasibility study comparing muscle ultrasound in patients with severe sepsis and healthy controls. We detected changes in muscle echogenicity in the early course of sepsis, while fasciculations evolved later. Muscle ultrasound is an easily applicable, non-invasive diagnostic tool for use in the intensive care setting and may facilitate bedside assessment of the muscle state in critically ill patients. Muscle ultrasound adds to neurophysiological testing knowledge with respect to morphological changes of muscles and may prove to be a screening tool for use prior to subjecting patients to more invasive techniques such as EMG and/or muscle biopsy. To date, the impact of muscle ultrasound on the prediction of functional outcome is unclear. Further studies are needed to describe the detailed time course of ultrasonic muscle changes and the evolvement of spontaneous activity in sepsis in relation to the functional clinical outcome. Here, a reliable differentiation between CIP and CIM (for example, using direct muscle stimulation and muscle biopsies) as well as a comparison between critically ill patients with and without ICUAW is required.

## Key messages

• Changes in muscle echogenicity can be detected early in the course of sepsis using muscle ultrasound.

• Muscle fasciculations are also detectable and evolve later.

• Muscle ultrasound might be a useful, easily applicable, non-invasive early screening tool prior to subjecting patients to more invasive techniques such as EMG and/or muscle biopsy.

## Abbreviations

CIM: Critical illness myopathy; CIP: Critical illness polyneuropathy; EMG: Electromyography; ICUAW: ICU-acquired weakness.

## Competing interests

The authors declare that they have no competing interests.

## Authors’ contributions

AG participated in study design, acquisition of data, analysis and interpretation of data, and also helped to draft the manuscript. UT participated in the acquisition of data and was involved in the analysis and interpretation of data. CP performed the statistical analysis, was involved in the analysis and interpretation of data, and helped to draft the manuscript. KL participated in the acquisition of data (medication, times of stay in the ICU, fluid balances, microorganisms) and was involved in the analysis and interpretation of data. JZ participated in the acquisition of data and was involved in the analysis and interpretation of data. OWW participated in the design of the study and was involved in the analysis and interpretation of data. FMB participated in the design of the study, was involved in the analysis and interpretation of data, and helped to draft the manuscript. HA obtained the funding, participated in the design of the study and in data acquisition, as well as being involved in the analysis and interpretation of data, and finally also helped to draft the manuscript. All authors read, critically revised and approved the final manuscript.

## Supplementary Material

Additional file 1 **Additional file 1 is a figure showing boxplots (left) and parallel coordinate plots (right) of nerve conduction measurements of patients.** Each patient is represented by a grey circle and measurements of the same patient over time are connected with a line. Local reference values (lower limit of normal) are given by the reference axis. The figure only shows the results of two motor nerves (CMAP = compound motor action potential) and one sensible nerve (SNAP = sensory nerve action potential) as typical examples. The measurements of the other nerves studied did not show divergent results. Click here for file
