# Supplementary material for: Muscle ultrasound for early assessment of critical illness neuromyopathy in severe sepsis
Source: Crit Care. 2013 Oct 7;17(5):R227. doi: 10.1186/cc13050 (PMC4057413; doi:10.1186/cc13050)
Supplement: Additional file 1 — is a figure showing boxplots (left) and parallel coordinate plots (right) of nerve conduction measurements of patients. Each patient is represented by a grey circle and measurements of the same patient over time are connected with a line. Local reference values (lower limit of normal) are given by the reference axis. The figure only shows the results of two motor nerves (CMAP = compound motor action potential) and one sensible nerve (SNAP = sensory nerve action potential) as typical examples. The measurements of the other nerves studied did not show divergent results. [file cc13050-S1.pdf]

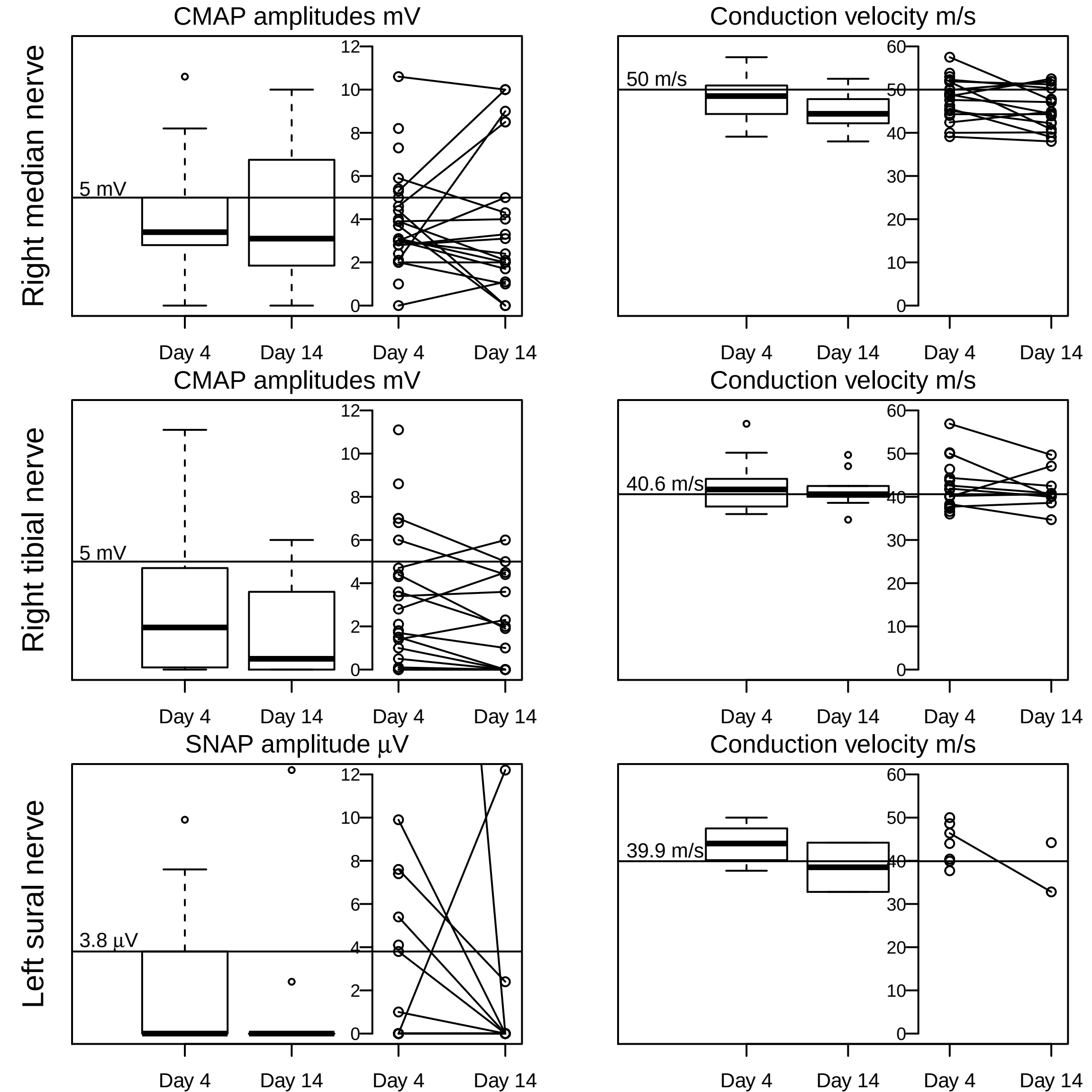

CMAP amplitudes mV
Conduction velocity m/s
Right median nerve
5 mV
50 m/s
CMAP amplitudes mV
Conduction velocity m/s
Right tibial nerve
5 mV
40.6 m/s
SNAP amplitude µV
Conduction velocity m/s
Left sural nerve
3.8 µV
39.9 m/s
Day 4
Day 14
Day 4
Day 14

**Appendix Figure** Boxplots (left) and parallel coordinate plots (right) of nerve conduction measurements of patients. Each patient is represented by a grey circle and measurements of the same patient over time are connected with a line. Local reference values (lower limit of normal) are given by the reference axis. The figure only shows the results of 2 motor (CMAP=compound motor action potential) and 1 sensible nerves (SNAP = sensory nerve action potential) as typical examples. The measurements of the other nerves studied did not show divergent results.
